# Bloom’s taxonomy-based comparison of artificial intelligence and dental students in restorative dentistry

**DOI:** 10.1186/s12909-026-09928-8

**Published:** 2026-07-13

**Authors:** Onur Akkurt, Ebru Yılmaz, Nilgün Akgül

**Affiliations:** Department of Restorative Dentistry, Faculty of Dentistry, Pamukkale University, Denizli, 20160 Türkiye

**Keywords:** Artificial intelligence, Dental education, Restorative dentistry, ChatGPT, Microsoft Copilot, Google Gemini, Bloom’s taxonomy

## Abstract

**Background:**

The aim of this study is to compare the performance of three large language models (ChatGPT 5, Microsoft Copilot, and Google Gemini 3), with that of dental students using their responses to multiple-choice questions (MCQs) in restorative dentistry. Accuracy of responses were analyzed across the knowledge and cognitive process dimensions of the revised Bloom’s taxonomy (RBT), as well as across subject areas.

**Methods:**

The restorative dentistry exam questions used in this study were drawn from Turkish Dentistry Specialization Entrance Exam (DUS) administered between 2020 and 2025. The 90 five-option, single-best-answer MCQs were classified according to the RBT to ensure cognitive diversity. Following the exclusion of one exam question which had been annulled by the examination authority, the data analysis was performed on the remaining 89 exam questions. Accuracy of AI models and dental students was compared using Pearson’s chi-square test and Monte Carlo-corrected Fisher’s exact test. Pairwise comparisons were carried out via Bonferroni-corrected Z-test. The results were presented as frequencies and percentages, and *p* < 0.050 was considered statistically significant.

**Results:**

Microsoft Copilot and Gemini 3 showed similar performance in answering MCQs, both models achieved higher accuracy than ChatGPT 5 and students (*p* < 0.001). ChatGPT 5’s overall accuracy was found to be significantly higher than that of the students. Accuracy of responses varied according to Bloom’s taxonomy levels and subject areas. Microsoft Copilot exhibited over 90% accuracy in all categories of Bloom’s knowledge and cognitive process dimensions. At the application level of the cognitive process dimension, all chatbots descriptively achieved 100% accuracy; however, this subgroup difference did not reach statistical significance. Chatbot performance was generally superior to that of students across the subject areas of adhesive dentistry, dentin hypersensitivity, dental caries, tooth whitening, aesthetic restorative procedures, contemporary restorative materials, preventive dentistry, lasers, and saliva.

**Conclusion:**

AI-based chatbots demonstrate considerable potential in answering questions about restorative dentistry. At the same time, the differences in performance observed among different models suggest there could be variation in the accuracy values of these systems depending on both the taxonomic level and the subject area of restorative dentistry. The findings support the potential use of chatbots as complementary learning resources in dental education. However, the reliability of such systems should be consistently verified and tested in both an academic and clinical setting under the direct supervision of experts. Moreover, students should be equipped with critical thinking skills to appropriately evaluate and use these systems.

## Introduction

Artificial intelligence (AI) is a multidisciplinary field, which focuses on designing and developing computer-based systems that are able to emulate cognitive processes characteristic of human intelligence, such as learning, logical reasoning, decision-making, and problem-solving [1–11]. Recent advances in computer processing power and in big-data analytics along with the development of deep learning (DL) algorithms are rapidly increasing the integration of AI applications into healthcare domains, including education, diagnostics, and clinical assessment. This technological transformation has led to dramatic increase in the use of intelligent tools that support healthcare professionals in acquisition, practice, and evaluation of clinical competence [1, 2].

Dialogue-based systems based on AI, typically called AI chatbots, represent one of the most common applications of AI technologies. These platforms are powered by large language models (LLMs), which, by virtue of their training on large-scale datasets, are capable of complex language understanding and generation tasks [1–3, 5, 6, 10, 11]. By virtue of their natural language processing capability, these tools can quickly respond to structured queries like multiple-choice questions (MCQs) [2, 5, 11]. In medical and dental disciplines, AI-supported chatbots are increasingly regarded as innovative support tools for both students and clinicians in theoretical instruction and clinical decision-making process [1–4]. Additionally, these systems are able to provide responses to queries posed in different languages [11, 12].

AI-based chatbots are used in many areas, including academic writing, education, the learning processes of medical and dental students, diagnostic support, and the provision of information about diseases [11, 13]. Therefore, comparing the performance of these systems with that of humans in dental education and clinical practice constitutes an important benchmark for evaluating the potential applications of AI in dentistry [11].

Restorative dentistry is one of the cognitively complex and demanding disciplines of the clinical assessment process. It covers a broad range of subject areas, including dental hard tissues, dental caries, adhesive dentistry, direct and indirect restorative procedures, restorative materials and their applications, polymerization methods, dentin hypersensitivity, bleaching, and preventive dentistry practices [1, 14]. Although numerous studies have evaluated the performance of AI-based chatbots on general medical and dental question sets, the number of studies have focused on specific dental subfields such as restorative dentistry remains limited [1, 3–6, 11, 15, 16].

Bloom and colleagues published in 1956 a taxonomy that classified educational objectives for the cognitive domain [17]. This taxonomy differentiates between different types and levels of learning according to the cognitive processes involved in learning. Although widely adopted by educators, the taxonomy has been progressively refined over time to address evolving instructional and assessment needs. Krathwohl [18] revised Bloom’s taxonomy to better meet the requirements of curriculum developers, teachers, and students. Subsequent studies have continued to refine and reinterpret the revised Bloom’s taxonomy (RBT) across different contexts [19–21].

The present study aims to compare the performance of three AI-based chatbots with that of dental students based on their responses to restorative dentistry questions drawn from the DUS examination administered between 2020 and 2025, and to assess the results on the basis of the RBT [18] and subject areas. DUS questions were selected because of their comprehensive structure, which robustly evaluates both domain-specific knowledge and clinical reasoning skills. Owing to scarcity of studies addressing this area from a taxonomic perspective in the existing literature [22], this study is expected to fill an important gap in the assessment of reliability of AI technologies in dental education. In this context, null hypothesis posits that there is no statistically significant difference in performance between the AI chatbots evaluated using 2020–2025 DUS restorative dentistry questions and dental students.

## Methods

### Ethical considerations and study design

Ethical approval for this study was obtained prior to commencement from the Non-Interventional Clinical Research Ethics Committee of Pamukkale University (E-60116787-020-872407). Participation was based on voluntariness, and informed consent was obtained from the students included in the study.

This study was designed to compare the accuracy of responses generated by the free-access web versions of three publicly accessible AI chatbot interfaces (ChatGPT 5, Microsoft Copilot, and Google Gemini 3) with those of dental students, using MCQs selected from the restorative dentistry section of the DUS. During data collection, all chatbot responses were obtained through the web interfaces of the respective platforms, rather than through application programming interfaces (APIs) or mobile applications. The displayed model labels at the time of testing, as shown in the user interface, were recorded as ChatGPT 5, Microsoft Copilot, and Google Gemini 3. The free-access tier was used for all three chatbot platforms throughout the testing period. No additional plugins, uploaded files, custom instructions, prompt engineering strategies, or contextual guidance were used. Web-search or retrieval functions, when available in the interface, were kept in their default settings and were not manually modified during testing. Because publicly accessible commercial LLM interfaces may be updated dynamically and may not always provide a stable API-style version string to end users, the model labels were reported as displayed in the user interface at the time of data collection.

For this purpose, the questions were entered into each chatbot in their original Turkish format, without translation into English. The reliability of data was ensured through cross-checking each of the responses against the official ÖSYM answer key, and through verification of the entire dataset by two specialists in restorative dentistry. This approach allowed for the assessment of AI chatbot performance across a large dataset spanning multiple examination years.

MCQs were entered manually into the AI-based chatbots using no other training data, prompt engineering, nor contextual guidance. To standardize the evaluation environment, identical question wording and testing conditions were applied across all models. Previous studies have shown that some LLMs may generate inconsistent responses to the same question when asked at different times or across repeated trials as well as generate multiple draft answers for a single query [3, 23]. To assess temporal consistency, each chatbot model was tested with a full set of questions set on three separate days at three different time points, resulting in data collected from nine sessions. In order to ensure consistency and to minimize variability, all of the questions were administered on March 15, 16, and 17, 2026, and each session was repeated three times. Prior to each session, the chat interface was reset so as to remove possible carryover from previous responses. For the AI chatbot groups, responses obtained across repeated testing sessions were included in the analysis as repeated outputs for each model. This repeated-testing approach was used to assess the temporal consistency of chatbot responses, as LLM outputs may vary across sessions because of non-deterministic response generation, dynamic interface behavior, and provider-side updates. In contrast, each student completed the questionnaire only once to avoid recall, learning, or test-familiarity effects that could occur with repeated human testing. Therefore, repeated AI outputs were not intended to represent independent human participants but rather to characterize model-level response stability over time. The outputs generated were exported to an Excel spreadsheet for subsequent analysis.

The study sample comprised 130 fourth-year students and 106 intern dentists from the Faculty of Dentistry at Pamukkale University. Each participant completed the questionnaire only once and did not provide multiple responses to the same item. Data on students’ examination performance were obtained from anonymized records of face-to-face administered questionnaires. No personally identifiable information was collected. Only demographic data, including age, gender, and academic year of study, were recorded. The dataset, including demographic variables and responses categorized as correct, incorrect, and unanswered, was subsequently transferred into an electronic database. All study procedures were conducted in accordance with the ethical principles of the Declaration of Helsinki [24].

### Question classification

The question set used in this assessment was classified through a consensus-based approach by two experienced faculty members (E.Y. and N.A.) in restorative dentistry. The two experts reviewed each question together and assigned the final Bloom’s taxonomy category and subject-area classification after discussion and agreement. Because the classifications were made through joint consensus rather than independent rating, an inter-rater agreement statistic such as Cohen’s kappa coefficient could not be calculated. The classification of questions according to Bloom’s Taxonomy was done along the dimensions of knowledge (factual and conceptual) and cognitive processes (remembering, understanding, applying, analyzing). Classification procedure according to the subject areas was carried out under the following headings: histology, physiology, and embryology of dental hard tissues; dental caries; non-carious lesions of dental hard tissues; saliva; dental cements; adhesive dentistry; preventive dentistry practices; composite resins; discoloration and bleaching in dentistry; dentine hypersensitivity and its treatment; amalgam; aesthetic restorative techniques; polymerization in dentistry; color in dentistry; indirect restorative techniques; lasers; pulp capping treatments; contemporary restorative materials; and tooth preparation.

### Inclusion and exclusion criteria for the data source

To ensure that the dataset was up to date, it was derived from 90 multiple-choice questions obtained from nine DUS sessions administered over the past five years. One question annulled by the examination authority was excluded from the statistical analysis. The inclusion criteria therefore comprised 89 MCQs related to restorative dentistry, written in Turkish, officially published, and designed with a single correct answer. Both text- and image-based questions were included in the study. For image-based questions, the original visual materials published in the official DUS question documents were presented directly to each chatbot together with the corresponding question stem and answer options. Thus, the visual content was provided as image input rather than being converted into a textual description. No optical character recognition (OCR), manual image transcription, or investigator-generated visual description was used. The same image, original Turkish question stem, and answer options were entered into all three chatbot platforms. The image-based questions were obtained from the official examination documents without additional editing, except for ensuring that the visual material, question stem, and answer options were clearly visible in the chatbot interface. Among the 89 MCQs included in the final analysis, 2 questions (2.2%) were image-based and 87 questions (97.8%) were text-only.

The inclusion criteria for student participants consisted of being enrolled at the Faculty of Dentistry of Pamukkale University, being a fourth- or fifth-year student in an educational program, having a level of clinical experience appropriate to the respective academic year, having sufficient language proficiency to comprehend the survey questions, and voluntarily participating after giving informed consent.

The exclusion criteria included enrollment in the first, second, or third year of study; submission of incomplete or prematurely terminated responses; insufficient language proficiency to comprehend the survey items; physical or cognitive limitations preventing participation; systematic inconsistencies in response patterns; and failure to provide informed consent.

### Sample size calculation

The sample size required in this study was calculated by performing a power analysis using the G*Power software version 3.1 with the chi-square test adopted for the comparison of accuracy of responses. Assuming a medium effect size (Cohen’s w = 0.25), an alpha level of 0.05, and a statistical power (1 − β) of 0.80, the required number of responses was calculated to be approximately 180. A total of 236 participants were included in the study, comprising fourth-year dental students and intern dentists. Additionally, the inclusion of all eligible question sets from 2020 to 2025 and their respective student cohorts enabled a more comprehensive evaluation of AI model performance and reduced potential bias associated with selective sampling.

### Statistical analysis

Data analysis was performed using R software version 4.5.0. Pearson’s chi-square test and the Monte Carlo-corrected Fisher’s exact test were utilized in order to compare the responses provided by the AI models and students. Pairwise comparisons were performed using the Bonferroni-corrected Z-test, and the results were presented as frequencies and percentages. Statistical significance was set at *p* < 0.050.

For chatbot models, each response generated in each repeated testing session was coded as correct or incorrect. Model-level accuracy was calculated by dividing the total number of correct responses by the total number of responses generated across all repeated sessions. No majority-vote aggregation or post hoc selection of chatbot responses was performed. Repeated chatbot outputs were retained to reflect response variability and temporal consistency across sessions. Because repeated outputs from the same chatbot model are not equivalent to independent responses from different human participants, chatbot–student comparisons were interpreted cautiously and were considered exploratory.

## Results

Upon evaluating overall AI chatbot performance, Microsoft Copilot (94.1%) and Gemini 3 (92.4%) reached the highest accuracy, with significantly higher than those observed for ChatGPT 5 and the students. Moreover, ChatGPT 5 achieved significantly higher accuracy than the students (*p* < 0.001).

Table 1 summarizes the responses of AI models and students according to Bloom’s knowledge dimension. A significant association was identified between the groups and the responses to questions at the factual knowledge level (*p* < 0.001). The percentages of incorrect answers were found to be 7.4% for ChatGPT 5, 3.2% for Microsoft Copilot, 3.7% for Gemini 3, and 19.1% for students. At the conceptual knowledge level, Microsoft Copilot and Gemini 3 demonstrated higher accuracy than ChatGPT 5 and the students.

**Table 1:** AI models vs students by Bloom’s taxonomy (knowledge)

Bloom level	Response	ChatGPT 5	Microsoft Copilot	Gemini 3	Students	Total	test statistic	*p*
L1 (Factual)	Wrong	14 (7.4) ^a^	6 (3.2) ^a^	7 (3.7) ^a^	946 (19.1) ^b^	973 (17.6)	73.333	**< 0.001** ^**x**^
	Correct	175 (92.6) ^a^	183 (96.8) ^a^	182 (96.3) ^a^	4010 (80.9) ^b^	4550 (82.4)		
L2 (Conceptual)	Wrong	86 (14.1) ^a^	41 (6.7) ^b^	54 (8.8) ^b^	2838 (17.7) ^a^	3019 (16.9)	84.406	**< 0.001** ^**x**^
	Correct	526 (86) ^a^	571 (93.3) ^b^	558 (91.2) ^b^	13,210 (82.3) ^a^	14,865 (83.1)		
Total	Wrong	100 (12.5) ^a^	47 (5.9) ^b^	61 (7.6) ^b^	3784 (18) ^c^	3992 (17.1)	146.852	**< 0.001** ^**x**^
	Correct	701 (87.5) ^a^	754 (94.1) ^b^	740 (92.4) ^b^	17,220 (82) ^c^	19,415 (82.9)		

^x^ Pearson chi-square test; ^a−c^ same letter: no significant difference; frequency (%)

Bold values indicate statistically significant test results (*p* < 0.05)

Table 2 shows the comparison of responses of students and AI models based on Bloom’s cognitive process dimensions. Significant associations were found at the remembering, understanding, and analyzing levels (*p* < 0.001, *p* < 0.001, and *p* = 0.002, respectively). At the application level, all chatbot models correctly answered all 18 items, whereas the students achieved an accuracy value of 87.3%; however, this subgroup comparison did not reach statistical significance (*p* = 0.052). At the understanding level, Microsoft Copilot and Gemini 3 performed significantly better than both ChatGPT 5 and the students. At the analyzing level, however, the accuracy of Gemini 3 was lower compared with the other chatbots and the students.

**Table 2:** AI models vs students by Bloom’s taxonomy (cognitive process)

Bloom level	Response	ChatGPT 5	Microsoft Copilot	Gemini 3	Students	Total	test statistic	*P*
L1 (Remembering)	Wrong	14 (6.5) ^a^	6 (2.8) ^a^	7 (3.2) ^a^	1037 (18.3) ^b^	1064 (16.9)	84.230	**< 0.001** ^**x**^
	Correct	202 (93.5) ^a^	210 (97.2) ^a^	209 (96.8) ^a^	4627 (81.7) ^b^	5248 (83.1)		
L2 (Understanding)	Wrong	83 (17.7) ^a^	38 (8.1) ^b^	37 (7.9) ^b^	2361 (19.2) ^a^	2519 (18.4)	73.096	**< 0.001** ^**x**^
	Correct	385 (82.3) ^a^	430 (91.9) ^b^	431 (92.1) ^b^	9911 (80.8) ^a^	11,157 (81.6)		
L3 (Applying)	Wrong	0 (0)	0 (0)	0 (0)	60 (12.7)	60 (11.4)	6.636	0.052^y^
	Correct	18 (100)	18 (100)	18 (100)	412 (87.3)	466 (88.6)		
L4 (Analyzing)	Wrong	3 (3) ^a^	3 (3) ^a^	17 (17.2) ^b^	326 (12.6) ^b^	349 (12.1)	21.633	**0.002** ^**y**^
	Correct	96 (97) ^a^	96 (97) ^a^	82 (82.8) ^b^	2270 (87.4) ^b^	2544 (87.9)		

^x^ Pearson chi-square test; ^y^ Monte Carlo-corrected Fisher’s Exact test; ^a−b^ same letter: no significant difference; frequency (%)

Bold values indicate statistically significant test results (*p *< 0.05)

In the image-based questions, all three chatbot models achieved 18/18 correct responses (100%), whereas students achieved 416/472 correct responses (88.1%). However, this difference did not reach statistical significance according to the Monte Carlo-corrected Fisher’s exact test (test statistic = 7.170, *p* = 0.060). In Bonferroni-corrected pairwise comparisons, no significant differences were observed between the chatbot models and students for image-based questions.

Table 3 presents the comparison of the responses from AI models and students according to subject areas. Statistically significant associations were identified between response distributions and groups in the subject areas of adhesive dentistry, dental cements, dentin hypersensitivity and its management–lasers, composite resins, and composite resins–polymerization in dentistry (*p* = 0.004, *p* = 0.014, *p* = 0.003, *p* = 0.045, and *p* = 0.021, respectively); however, no significant differences were detected in pairwise comparisons. Significant associations among the groups were determined in the subject areas of dental caries, polymerization in dentistry, color in dentistry, discoloration and bleaching in dentistry, non-carious lesions of dental hard tissues, preventive dentistry practices, pulp capping treatments, and saliva (*p* < 0.001, *p* = 0.001, *p* = 0.002, *p* < 0.001, *p* < 0.001, *p* < 0.001, *p* < 0.001, and *p* < 0.001, respectively). For color in dentistry, students attained higher accuracy than all chatbot models, while in other subject areas, their accuracy was comparable or lower.

**Table 3:** AI models vs students by subject area

Subject area	Response	ChatGPT 5	Microsoft Copilot	Gemini 3	Students	Total	test statistic	*p*
Adhesive dentistry	Wrong	3 (6.7)	0 (0)	0 (0)	135 (11.4)	138 (10.5)	15.009	**0.004** ^**x**^
	Correct	42 (93.3)	45 (100)	45 (100)	1045 (88.6)	1177 (89.5)		
Amalgam	Wrong	6 (13.3)	6 (13.3)	3 (6.7)	140 (11.9)	155 (11.8)	1.342	0.734^x^
	Correct	39 (86.7)	39 (86.7)	42 (93.3)	1040 (88.1)	1160 (88.2)		
Dental cements	Wrong	6 (22.2)	0 (0)	0 (0)	62 (8.8)	68 (8.6)	9.993	**0.014** ^**x**^
	Correct	21 (77.8)	27 (100)	27 (100)	646 (91.2)	721 (91.4)		
Dental cements - composite resin	Wrong	0 (0)	0 (0)	0 (0)	44 (18.6)	44 (16.7)	4.382	0.083^x^
	Correct	9 (100)	9 (100)	9 (100)	192 (81.4)	219 (83.3)		
Dentin hypersensitivity and its treatment	Wrong	3 (8.3)	3 (8.3)	0 (0)	104 (11)	110 (10.5)	5.331	0.180^x^
	Correct	33 (91.7)	33 (91.7)	36 (100)	840 (89)	942 (89.5)		
Dentin hypersensitivity and its treatment - lasers	Wrong	0 (0)	0 (0)	0 (0)	78 (33.1)	78 (29.7)	12.928	**0.003** ^**x**^
	Correct	9 (100)	9 (100)	9 (100)	158 (67)	185 (70.3)		
Dental caries	Wrong	7 (9.7) ^a^	7 (9.7) ^a^	0 (0) ^b^	309 (16.4) ^a^	323 (15.4)	24.661	**< 0.001** ^**x**^
	Correct	65 (90.3) ^a^	65 (90.3) ^a^	72 (100) ^b^	1579 (83.6) ^a^	1781 (84.7)		
Polymerization	Wrong	16 (22.2) ^a^	1 (1.4) ^b^	9 (12.5) ^ab^	418 (22.1) ^a^	444 (21.1)	28.449	**0.001** ^**x**^
	Correct	56 (77.8) ^a^	71 (98.6) ^b^	63 (87.5) ^ab^	1470 (77.9) ^a^	1660 (78.9)		
Color	Wrong	8 (29.6) ^ab^	6 (22.2) ^ab^	12 (44.4) ^a^	116 (16.4) ^b^	142 (18)	14.783	**0.002** ^**x**^
	Correct	19 (70.4) ^ab^	21 (77.8) ^ab^	15 (55.6) ^a^	592 (83.6) ^b^	647 (82)		
Discoloration/ bleaching	Wrong	8 (8.1) ^a^	0 (0) ^b^	8 (8.1) ^a^	538 (20.7) ^c^	554 (19.2)	59.323	**< 0.001** ^**x**^
	Correct	91 (91.9) ^a^	99 (100) ^b^	91 (91.9) ^a^	2058 (79.3) ^c^	2339 (80.9)		
Tooth preparation	Wrong	3 (33.3)	0 (0)	0 (0)	42 (17.8)	45 (17.1)	4.602	0.107^x^
	Correct	6 (66.7)	9 (100)	9 (100)	194 (82.2)	218 (82.9)		
Dental hard tissues	Wrong	0 (0)	4 (22.2)	1 (5.6)	60 (12.7)	65 (12.4)	4.565	0.155^x^
	Correct	18 (100)	14 (77.8)	17 (94.4)	412 (87.3)	461 (87.6)		
Dental hard-tissue histology, physiology, and embryology	Wrong	19 (26.4)	14 (19.4)	15 (20.8)	460 (24.4)	508 (24.1)	1.439	0.679^x^
	Correct	53 (73.6)	58 (80.6)	57 (79.2)	1428 (75.6)	1596 (75.9)		
Non-carious lesions	Wrong	0 (0) ^a^	6 (22.2) ^b^	9 (33.3) ^b^	131 (21.6) ^ab^	146 (16.9)	12.961	**< 0.001** ^**x**^
	Correct	27 (100) ^a^	21 (77.8) ^b^	18 (66.7) ^b^	649 (78.4) ^ab^	715 (83.1)		
Aesthetic restorations	Wrong	0 (0)	0 (0)	0 (0)	7 (3)	7 (2.7)	0.498	1.000^x^
	Correct	9 (100)	9 (100)	9 (100)	229 (97)	256 (97.3)		
Contemporary materials	Wrong	0 (0)	0 (0)	0 (0)	41 (17.4)	41 (15.6)	3.771	0.129^x^
	Correct	9 (100)	9 (100)	9 (100)	195 (82.6)	222 (84.4)		
Indirect restorations	Wrong	2 (22.2)	0 (0)	0 (0)	54 (22.9)	56 (21.3)	4.646	0.166^x^
	Correct	7 (77.8)	9 (100)	9 (100)	182 (77.1)	207 (78.7)		
Composite resins	Wrong	5 (18.5)	0 (0)	0 (0)	80 (11.3)	85 (10.8)	8.878	**0.045** ^**x**^
	Correct	22 (81.5)	27 (100)	27 (100)	628 (88.7)	704 (89.2)		
Composite resins - polymerization	Wrong	0 (0)	0 (0)	0 (0)	78 (16.5)	78 (14.8)	10.489	**0.021** ^**x**^
	Correct	18 (100)	18 (100)	18 (100)	394 (83.5)	448 (85.2)		
Preventive dentistry	Wrong	2 (3.7) ^a^	0 (0) ^a^	0 (0) ^a^	361 (25.5) ^b^	363 (23)	67.621	**< 0.001** ^**x**^
	Correct	52 (96.3) ^a^	54 (100) ^a^	54 (100) ^a^	1055 (74.5) ^b^	1215 (77)		
Lasers	Wrong	0 (0)	0 (0)	0 (0)	51 (10.8)	51 (9.7)	4.874	0.079^x^
	Correct	18 (100)	18 (100)	18 (100)	421 (89.2)	475 (90.3)		
Pulp capping	Wrong	12 (66.7) ^a^	0 (0) ^b^	0 (0) ^b^	90 (19.1) ^b^	102 (19.4)	29.220	**< 0.001** ^**x**^
	Correct	6 (33.3) ^a^	18 (100) ^b^	18 (100) ^b^	382 (80.9) ^b^	424 (80.6)		
Saliva	Wrong	0 (0) ^a^	0 (0) ^a^	4 (5.6) ^a^	391 (20.7) ^b^	395 (18.8)	65.754	**< 0.001** ^**x**^
	Correct	72 (100) ^a^	72 (100) ^a^	68 (94.4) ^a^	1497 (79.3) ^b^	1709 (81.2)		

^x^ Monte Carlo-corrected Fisher’s exact test; ^a−c^ same letter: no significant difference; frequency (%)

Bold values indicate statistically significant test results (*p* < 0.05)

## Discussion

Over the past several years, the accuracy and comprehensiveness of responses generated by LLMs have been increasingly investigated in scientific research. AI and chatbot systems that are continuously developed have become an important focus of research in both health education and clinical practice [4]. The present study comparatively evaluated the responses of three free and up-to-date LLM-based chatbots to restorative dentistry questions from the DUS examination against those of dental students. The evaluation was not limited to overall accuracy; instead, the questions were also analyzed in terms of the knowledge and cognitive process dimensions of Bloom’s taxonomy and categorized by subject areas. Additionally, image-based questions were incorporated into the analysis, and the method used to present visual content to the chatbot interfaces was clarified in the Methods section. However, because only 2 of the 89 included MCQs were image-based, findings related to image-containing items should be interpreted cautiously. All models achieved an overall value of accuracy above 87.5%. Differences were observed across subject areas categories. In non-carious lesions of dental hard tissues, ChatGPT 5 performed significantly better; Microsoft Copilot exhibited superior performance in polymerization and discoloration/bleaching procedures; and Gemini 3 achieved better results than the other models and students in dental caries. Furthermore, analyses based on the RBT demonstrated statistically significant differences between the performance of AI chatbots and students. Therefore, the null hypothesis was rejected.

The chatbots tested in this study differ in terms of their AI architecture and data-processing capacities. Despite facilitating access to scientific knowledge, this diversity also necessitates rigorous evaluation of the validity and reliability of the models used by students [25]. These results reveal both potential applications and the inherent strengths and limitations of AI chatbots in dentistry. Moreover, in the domain of color, where students outperformed the chatbots in terms of accuracy, the results further support the need for critical appraisal of AI-generated responses within specific areas of dentistry.

The original purpose of developing AI-based LLMs was not to resolve medical and dental problems, which could limit their ability to interpret the complexities of many clinical situations [26]. The performance of AI-based chatbots in responding to medical and dental questions has been extensively studied in the literature [4, 27–29]. reported an accuracy of 89% for ChatGPT-4o across several medical licensing examinations and highlighted substantial variation in performance across specialties. Uehara et al. [28] found that both ChatGPT-3.5 and ChatGPT-4o exhibited higher accuracy in foundational medical sciences which included pathology, pharmacology, and microbiology, on the Japanese National Dental Examination. Chau et al. [29] demonstrated that ChatGPT-4o exceeded the passing threshold in U.S. and U.K. dental licensing examinations, whereas ChatGPT-3.5 failed to meet this threshold. In a study on dentomaxillofacial radiology, ChatGPT-4o showed the highest accuracy, while Microsoft Copilot exhibited lower performance [30]. Another international study covering the USMLE, PLAB, and medical licensing examinations in Hong Kong and China also found that GPT-4o outperformed other LLMs [31]. In Turkish prosthodontics examinations, Gemini and ChatGPT-3.5 achieved comparable, albeit relatively low, levels of accuracy [32]. Conversely, Sarı et al. [4] reported overall accuracies of 84.0% for ChatGPT-4o and 81.8% for Gemini Advanced, with both models achieving above-average performance in restorative dentistry. A study conducted by Eraslan et al. [33] used five different AI chatbots and 126 prosthodontic-type questions to evaluate their use. They found that the highest performance was shown by Microsoft Copilot at an accuracy of 73%. In the present study, Microsoft Copilot and Gemini 3 reached greater overall accuracy than ChatGPT 5. Possible explanations for these differences may include variations in information retrieval strategies, retrieval-augmented generation, and the representation of domain-specific knowledge in training data. However, these mechanisms were not directly evaluated in the present study; therefore, such explanations should be regarded as hypotheses rather than empirically demonstrated findings.

Although the high accuracy of responses obtained in our study share certain similarities with previous reports, differences emerged with regard to the ranking among chatbots and topic-specific performance patterns. The literature review revealed that only a limited number of studies have evaluated the performance of the free and up-to-date versions of ChatGPT 5 and Gemini 3 in dental subdisciplines. Therefore, further research evaluating the performance of these models across different dental specialties and question types is warranted.

LLMs are rapidly evolving and continuously updated AI technologies. Therefore, performance differences among chatbots are to be expected, and the results observed in this study may not fully reflect the performance of future versions. Although statistical differences were observed among the models, students generally exhibited lower levels of accuracy than most of the chatbots. These differences may reflect several possible factors, including variation in the representation of specialized knowledge within training data, differences in the coverage of interdisciplinary topics, and the varying complexity of subject areas. Similarly, the observed decline in accuracy from factual to conceptual knowledge may suggest differences in how chatbot models handle different types of knowledge; however, the mechanisms underlying these patterns were not directly examined in the present study. Therefore, these interpretations should be considered explanatory hypotheses rather than confirmed mechanisms.

In their comparative analysis, Rahman et al. [34] found that each chatbot possessed distinct strengths. ChatGPT was particularly recognized for its multimodal capabilities, whereas Gemini excelled in integrating and processing text, code, and visual inputs within a unified framework. In our study, however, three free-version chatbots were tested using structured MCQs, and their response accuracies were compared. Within this context, the overall performance of ChatGPT 5 found to be lower than that of Microsoft Copilot and Gemini 3.

Within Bloom’s taxonomy, factual knowledge addresses the question of “what,” whereas conceptual knowledge addresses the questions of “why” and “how” [18]. Şişmanoğlu et al. [11] classified the 2020–2021 DUS questions in terms of low and high difficulty levels and reported that accuracy of chatbot was decreasing with increasing taxonomic levels. In the present study, Microsoft Copilot performed similarly to the other chatbots at the factual knowledge level but achieved a higher accuracy at the conceptual knowledge level. Differences between students and AI chatbots were observed across several stages of Bloom’s cognitive process classification; however, the application-level comparison should be interpreted descriptively because it did not reach the pre-specified threshold for statistical significance. Copilot had an overall accuracy of above 91% across all levels of the cognitive process dimension. These findings suggest that chatbots may have potential beyond simple information recall; however, interpretations regarding higher-order cognitive levels, particularly the application level, should be made cautiously because not all subgroup comparisons reached statistical significance. Nevertheless, the mechanisms responsible for chatbot performance at higher levels of Bloom’s taxonomy were not examined in the present study. Consequently, any conclusions regarding the contribution of retrieval-augmented generation (RAG), online resource use, or in-model reasoning should be drawn with caution.

Our study differs from previous research in that it evaluated AI chatbots not only in terms of overall restorative dentistry but also at the level of individual subject areas. Variations in chatbot performance, as documented in discipline-specific studies, were also observed across subject areas in this study. Restorative dentistry has a wide scope, including structured clinical procedures, complex decision-making, and preventive approaches. This likely explains the differences in performance observed across subject areas. Therefore, the scope and diversity of training datasets play an important role in determining how reliable AI models are in different dental subdisciplines [1, 35].

However, the subject-area findings should be interpreted cautiously because some thematic categories included a limited number of questions. In such categories, a single correct or incorrect response could substantially affect the percentage accuracy, and therefore 100% accuracy should not necessarily be interpreted as true superiority of a given chatbot model.

The findings have notable implications for restorative dentistry practice and dental education. The high accuracy of LLMs suggests their potential as complementary tools in examination preparation, facilitating rapid and structured access to information. However, their relatively lower performance at specific taxonomic levels and in certain subject areas highlights the need for cautious and supervised use. Nevertheless, the higher accuracy achieved by chatbot models in this MCQ-based assessment should not be interpreted as evidence of superior overall clinical competence compared with dental students. Multiple-choice questions primarily evaluate declarative knowledge and selected aspects of clinical reasoning, whereas broader clinical competencies, including patient communication, treatment planning, risk assessment, and real-world clinical decision-making, were not assessed in this study.

From the perspective of dental practitioners, the findings suggest that AI-based chatbots may contribute to the efficient acquisition of knowledge and the summarization of clinical guidelines [4]. However, these limitations underscore the fact that evidence-based decision-making and clinical judgment remain central to clinical practice. Therefore, LLMs should be regarded as promising complementary resources, rather than substitutes for core educational resource, clinical experience, or professional expertise [36].

The results from the perspective of dental students can be interpreted through the lens of existing pedagogical approaches. The use of AI chatbot applications may strengthen retrieval practice as well as allow students to retest their knowledge in subject areas and question types they find challenging. Additionally, chatbots may structure the learning process in restorative dentistry, where case interpretation and terminology are closely intertwined, by providing step-by-step explanations. When employed in a controlled manner, such systems may also help reduce cognitive load by providing immediate feedback during self-directed learning [33].

Integrating AI literacy into a dental curriculum would allow students to use these tools as an aid in fulfilling their professional responsibilities and in striking a balance between efficiency and critical thinking. Nonetheless, several risks associated with the use of technology remain unaddressed. Over-reliance on chatbots may weaken students’ independence problem-solving skills, while disparities in access to advanced AI systems may further exacerbate educational inequalities across institutions [4]. For this reason, the present study evaluated chatbots that are freely accessible to students.

One of the main limitations of this study is that all questions were written in Turkish and based on a single examination format. The accuracy values achieved by an AI model may be affected by both the input language and the type of question asked. Although previous studies have reported similar outcomes for examinations administered in multiple languages, the generalizability of these findings remains limited [12]. Limitations also exist with regard to the limited number of image-based questions, the exclusive use of MCQs, and the limited coverage of specific Bloom’s taxonomy levels and subject areas. Moreover, the MCQ-based design primarily assessed declarative knowledge and selected aspects of clinical reasoning; therefore, broader clinical competencies such as patient communication, treatment planning, risk assessment, and real-world clinical decision-making were not evaluated.

Furthermore, because of the rapidly evolving nature of AI chatbots, the versions assessed in this study may differ from more advanced versions released in the future. Changes in training data, update frequency, and model architecture may affect performance and limit direct comparisons among systems. In addition, because this study used free-access public web interfaces rather than fixed API endpoints, exact backend model behavior may have been influenced by provider-side updates or routing decisions that were not fully visible to end users. Therefore, although the displayed model labels, access tiers, interface types, and retrieval settings were documented to improve reproducibility, model-versioning uncertainty remains a limitation, and the findings should be interpreted within the constraints of dynamic commercial AI systems.

Another limitation is that AI-generated responses were obtained through repeated testing of the same question set, whereas each student completed the questionnaire only once. This design reflects the different response dynamics of LLM-based systems and human participants: repeated chatbot testing was used to evaluate temporal consistency, whereas repeated student testing could introduce recall or learning effects. Nevertheless, repeated chatbot outputs may not fully satisfy the independence assumption underlying conventional group comparisons and may have increased the apparent precision of chatbot performance estimates. Therefore, comparisons between chatbot and student performance should be interpreted cautiously, and broad generalizations from sub-group analyses should be avoided.

In addition, because the Bloom’s taxonomy classifications were determined through expert consensus rather than independent rating, inter-rater agreement statistics could not be reported.

A further limitation is the potential for data leakage. Because the DUS questions used in this study were obtained from official examinations administered between 2020 and 2025 and are publicly available, it cannot be excluded that identical or highly similar questions may have been included in the training data of the evaluated AI models or accessed through retrieval-enabled systems. Therefore, the observed performance may not exclusively reflect the reasoning ability of the chatbots but may also partly reflect recognition of previously published examination content. Future studies using newly developed, non-public, or prospectively validated question sets may provide a more robust assessment of the reasoning capabilities of AI-based chatbots in restorative dentistry.

## Conclusions

This study is among the investigations that comparing multiple AI-based systems with dental students in restorative dentistry and evaluating the results using Bloom’s taxonomy and subject areas. The results indicate that Microsoft Copilot and Gemini 3 achieved higher overall accuracy than ChatGPT 5 in this MCQ-based assessment, and that all three chatbot models achieved higher accuracy than the dental students. However, these findings should be interpreted within the limits of a multiple-choice examination format and should not be considered evidence of superior overall clinical competence. However, subgroup findings, particularly those related to the application level of Bloom’s cognitive process dimension, should be interpreted with caution when statistical significance was not reached. The overall level of accuracy of AI-based chatbots’ responses supports their potential value as complementary tools in clinical education and exam preparation. However, differences in model performance as well as varying degrees of success in subject-specific areas may indicate that such technology needs to be chosen on user requirements and specific contextual factors. Therefore, future research should assess the applicability of these findings to other dental specialties, various languages, and multiple assessment frameworks for the purpose of supporting evidence-based integration of AI into health professions education.

## Data Availability

The data that support the findings of this study are available on request from the corresponding author. The data are not publicly available due to privacy or ethical restrictions.

## Abbreviations

AI: Artificial intelligence
DL: Deep learning
LLMs: Large language models
MCQs: Multiple-choice questions
RBT: Revised Bloom’s taxonomy
DUS: Turkish Dentistry Specialization Entrance Exam
ÖSYM: Assessment, Selection and Placement Center
